# A bidirectional 2-sample Mendelian randomization study of depressed mood and premature ovarian insufficiency

**DOI:** 10.1097/MD.0000000000048039

**Published:** 2026-03-13

**Authors:** Kan Chen, Li Wan, Xinmei Wang, Xiaorong Ma, Yanru Chen, Lu Han

**Affiliations:** a Xinjiang Uygur Autonomous Region Hospital of Traditional Chinese Medicine, Urumqi Xinjiang Uygur Autonomous Region, China; b Nursing School, Xinjiang Medical University, Urumqi, Xinjiang Uygur Autonomous Region, China.

**Keywords:** causality, depressed mood, Mendelian randomization, premature ovarian insufficiency

## Abstract

This study aimed to investigate the bidirectional causal relationship between depressed mood and premature ovarian insufficiency (POI) using a Mendelian randomization (MR) method. Genome wide association study data on depressed mood and POI were obtained using the IEU Open genome wide association study website. Closely related and independent single nucleotide polymorphisms (SNPs) were screened as instrumental variables (IVs) from depressed mood according to preset thresholds, and the association between depressed mood and the risk of developing POI was mainly assessed using inverse-variance weighted (IVW); the heterogeneity of SNPs was also assessed using CochranQ test. The MR-PRESSO test was used to detect the presence of outlier SNPs, and the MR-Egger intercept test was used to test the horizontal pleiotropy of SNPs. A “leave-one-out” sensitivity analysis was performed to test whether the MR results were influenced by a single SNP, and a 2-way MR analysis was performed by interchanging the screening processes for depressed mood and POI. The results of MR analysis indicated that there was no causal relationship between depressed mood and the occurrence of POI (odds ratio = 0.601, 95% confidence interval: 0.105–3.430, *P* = .566), and in the reverse MR analysis, there was no significant causal relationship between POI and depressed mood (odds ratio = 0.999, 95% confidence interval: 0.996–1.003, *P* = .663), none of the instrumental variables in the bidirectional MR analysis showed horizontal pleiotropy and heterogeneity. Genetics-based MR analysis found no significant causal relationship between depressed mood and POI.

## 1. Introduction

Premature ovarian insufficiency (POI) is a common gynecologic reproductive endocrine disorder characterized by the decline or interruption of ovarian function before the age of 40 years, which is often characterized by menstrual abnormalities, decreased fertility, and symptoms similar to those of menopause, but unlike menopause, it is a potentially delayed or reversible ovarian condition, with approximately 4% to 10% of women with POI being able to conceive naturally, and approximately 20% having successful ovulation induction.^[1]^ In addition, osteoporosis, cardiovascular disease, and cognitive decline are some of the long-term complications due to long-term estrogen deficiency. POI is a complex disease, and its multifactorial etiology and pathogenesis are not fully understood. Autoimmune factors, as well as genetic, medical, and affective-related factors, have been reported to be one of the potential causes of POI,^[2]^ with a global prevalence of about 1% to 2.8%,^[3,4]^ and even about 0.1% in women under 30 years of age,^[5]^ which seriously affects the quality of women lives and their physical and mental health. As half of the sky in modern society, women are not only the creators of material wealth, but also the promoters of social civilization, but with the increase of social pressure, more and more of them are accompanied by abnormalities of depressive manifestations, and as many as 43% of POI patients have depressive manifestations, which not only originate from the uncomfortable experience caused by the condition itself, but also include the treatment effect that is less than expected, lack of humanistic care, etc. Long-term negative psychological state interacts with the disease to form a vicious cycle, causing patients’ conditions to recur and further aggravating the progression of the disease.^[6–8]^ To date, some studies have found that in the treatment of patients with POI, emotional regulation is conducive to the recovery of patients with POI,^[9]^ but some studies have also shown that depressed mood may not increase in POI,^[10]^ although the current relevant results point out that there may be a close correlation between depressed mood and POI, it does not indicate a causal relationship between the 2, due to its privacy, and the relevant studies are all traditional Mendelian randomization offers the possibility of inferring causal associations between exposures and outcomes due to its intimate nature and the fact that studies are traditional observational epidemiological studies, which usually have limited sample sizes and are susceptible to confounders and reverse causality.MR is a statistical method based on whole-genome sequencing data and Mendel second law, which overcomes the limitations of observational studies and is used to uncover causal relationships.^[11]^ The core idea is to infer the causal relationship between exposure and outcome based on genetic variation, i.e., single nucleotide polymorphisms, as an instrumental variable. Since alleles in the parental generation are passed on to the offspring during meiosis according to the principle of “random assignment,” the MR method has been called The MR method is therefore called a “natural randomized controlled trial” and is not subject to the confounding factors and reverse causality found in traditional epidemiological studies.^[12]^ With the development of society, medicine has gradually shifted to a biomedical-social model, and people are paying more attention to the interplay between social and psychological factors and diseases. To further explore the link between depressed mood and POI, this study intends to investigate the bidirectional causal association between depressed mood and POI by MR method.

## 2. Data and methods

### 2.1. Research principles

**Figure 1.**
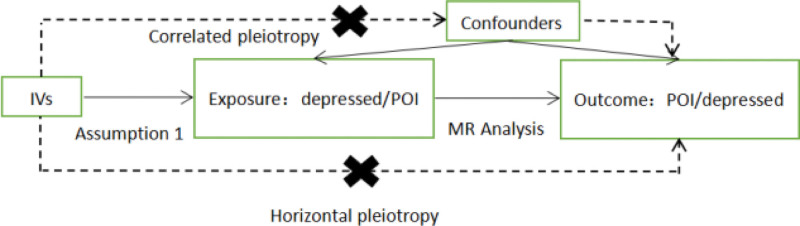
Model of the 2-sample MR analysis. MR = Mendelian randomization.

The rationale for MR is to use the genetic variation associated with exposure and outcome as an instrumental variable to infer whether there is a causal association between the 2. In this paper, depressed mood was used as exposure factor, SNPs significantly correlated with POI was used as IVs, and the outcome variable was POI. Meanwhile, exposure and outcome were interchanged in the bidirectional analysis. After the outliers were eliminated, the 2-sample MR 2-way analysis method was used for causality analysis, and the heterogeneity test and pleiotropic test were carried out. Finally, the reliability of the results was tested. The basic steps include: obtaining genome wide association study (GWAS) summary data, SNP screening and evaluation, statistical analysis and quality inspection. The accuracy of MR analysis is based on the satisfaction of the following 3 core assumptions^[13]^: IVs must be closely related to exposure, IVs was not associated with confounding factors affecting “expose-outcome”, and IVs only affected the outcome through exposure but not through other means, as shown in Fig. 1.

### 2.2. Data sources

Depressed mood with POI is not complete data are derived from the IEU open GWAS database (https://gwas.mrcieu.ac.uk/). The sample size of depressed data was 357,957 with 10,828,862 SNPs. The sample size of the study on POI was 118,482, including 254 POI patients and 118,228 controls, with a total of 16,379,677 SNPs. The above data sources were all from Europe. See Table 1 for details.

**Table 1:** Summary of the GWAS included in this 2-sample MR study.

Name	Sample size	No. of SNPs	Race	Gender	Year
Depressed	357,957	10,828,862	European	Male and female	2018
POI	118,482	16,379,677	European	Male and female	2021

GWAS = genome wide association study, MR = Mendelian randomization, POI = premature ovarian insufficiency, SNPs = single nucleotide polymorphisms.

### 2.3. Screening of IVs

IVs screening was performed according to the core assumptions of MR. Setting *P* < 5 × 10^-8^ as the screening condition, SNPs significantly associated with exposure were extracted from the GWAS summary database to satisfy the association hypothesis. The TwoSampleMR package of the R4.2.2 software was used for clump calculation, and parameters *r*^2^ = 0.001 and kb = 10,000 were set to exclude the influence of linkage imbalance and ensure the independence of IVs. If the number of SNPs is too small, parameters *P* < 5 × 10^-6^, *r*^2^ = 0.001 were set. kb = 10,000 for calculation.^[14]^ The above SNPs closely related to depressed were extracted from the GWAS data of POI, and the exposure and outcome data were corrected in the same direction to remove the SNPs of palindromic structure, and the 2 were collated and combined. MR-PRESSO was used to perform outlier test to remove horizontal pleotropy,^[15]^ delete outliers and eliminate SNPs directly related to POI.^[16]^

### 2.4. Statistical analysis

### 2.5. Estimation of causal effects

In this study, inverse-variance weighted (IVW), MR-Egger regression method, Weighted median method, Weighted mode method, and Simple mode method were adopted to estimate the causal effect between exposure and outcome variables.^[17]^ IVW method is mainly used for causality estimation, which is considered as the standard method for MR Analysis. When all the selected SNPs are valid IVs, IVW can provide accurate estimates. The principle is to combine Wald ratio estimates of each instrumental variable in causal estimation, and use random effects model if there is heterogeneity. When there is no heterogeneity, the fixed-effect model is adopted.^[15]^ The MR-Egger method can detect potential pleiotropy.^[18]^ The weighted median method requires more than half of the instrumental variables to be valid SNPs.^[19]^ The weighted model method can still provide a consistent estimate of causal effect when the invalid IVs is up to half.^[20]^ The simple model method can group SNPs with similar effects according to whether the estimated causal effects are similar.^[13]^

### 2.6. Quality control

In order to check the stability and reliability of MR Results, this study applied a variety of methods for quality control. First, the *F* value of a single SNPs was calculated respectively, and the weak IVs bias test was performed on the selected IVs. *F* = *β*^2^ exposure/SE^2^ exposure, *β* is the allele effect value of exposure, SE is the standard error of exposure.^[21]^ Second, Cochran Q test was used to evaluate the heterogeneity of SNPs. If the Cochran Q test was statistically significant, it indicated that there was significant heterogeneity in the analysis results. Thirdly, the MR-Egger intercept test is used to check whether SNP has pleiotropy. If the MR-Egger test has statistical significance, it indicates that the analysis results have significant pleiotropy. Fourth, Mendelian random polymorphism residuals and outliers are used to search for outlier SNPs in the results. If so, they are removed and re-analyzed. Fifth, the robustness of the results was tested by applying the “leave one method” to evaluate the effect of individual SNPs on the association between exposure and outcome variables by calculating the combined effect of the remaining SNPs after removing SNPs one by one. In this study, MR Analysis and quality control were performed using TwoSampleMR of R4.2.2 software, and the test level *α* = 0.05.

## 3. Results

### 3.1. MR results of depressed and POI

#### 3.1.1. Screened IVs

A total of 61 SNPs were screened out to be significantly correlated with depressed, and the POI summary data were retrieved and extracted, among which 1 SNP was missing from the POI GWAS data, and 2 palindromic sequences were excluded. For rs209156 and rs7827176, no outlier value was obtained by MR-PRESSO test. A total of 58 SNPs were finally included, and the *F*-statistic value of these SNPs was all >10, indicating that the possibility of weak variables was small.

### 3.2. MR results for both samples

**Figure 2.**
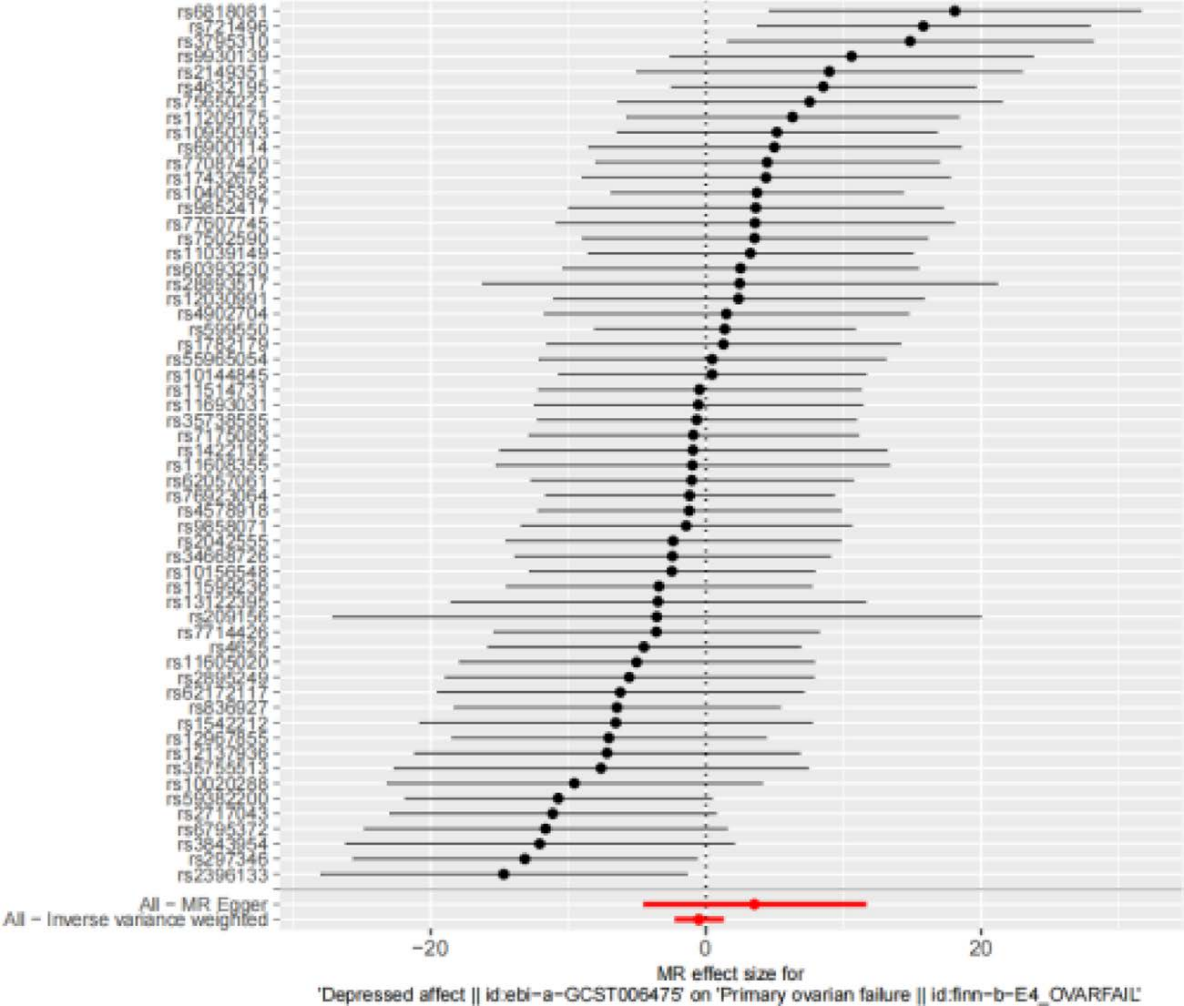
The MR analysis results for the associations from depressed affect to POI using 2 different methods: IVW and MR Egger. IVW = inverse-variance weighted, MR = Mendelian randomization, POI = .

**Figure 3.**
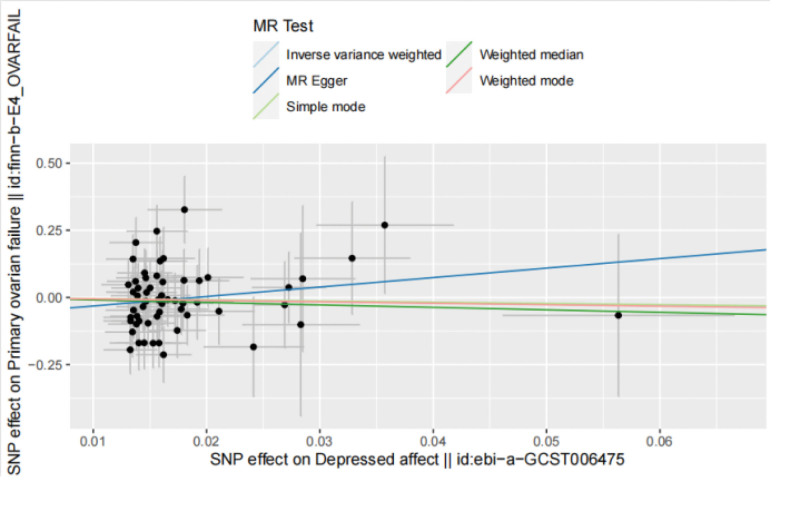
A scatter plot of the analysis results from depressed affect to POI using five different MR methods, with different colors representing different methods and dots representing different SNPs. MR = Mendelian randomization, POI = premature ovarian insufficiency, SNPs = single nucleotide polymorphisms.

IVW result: odds ratio (OR) = 0.601, 95% confidence interval (CI): 0.105 to 3.43E+00, *P* = .566, indicating that depressed may have nothing to do with the occurrence of POI. The forest diagram of MR analysis is shown in Fig. 2, and the MR-Egger method, as a supplement to the IVW results, also indicates that there is no obvious causal relationship between depressed and POI. The relevant results of the 5 MR testing methods are shown in Table 2, and the scatter diagram is shown in Fig. 3.

**Table 2:** MR analysis from depressed affect to POI.

Method	nSNP	OR (95% CI)	*P*-value
MR Egger	58	33.886 (0.011–1.08E+05)	.395
Weighted median	58	0.398 (0.038–4.14E+00)	.441
Inverse variance weighted	58	0.601 (0.105–3.43E+00)	.566
Simple mode	58	0.641 (0.003–118E+02)	.868
Weighted mode	58	0.582 (0.004–9.45E+01)	.836

CI = confidence interval, MR = Mendelian randomization, OR = odds ratio, POI = premature ovarian insufficiency, SNPs = single nucleotide polymorphisms.

### 3.3. Quality control

**Figure 4.**
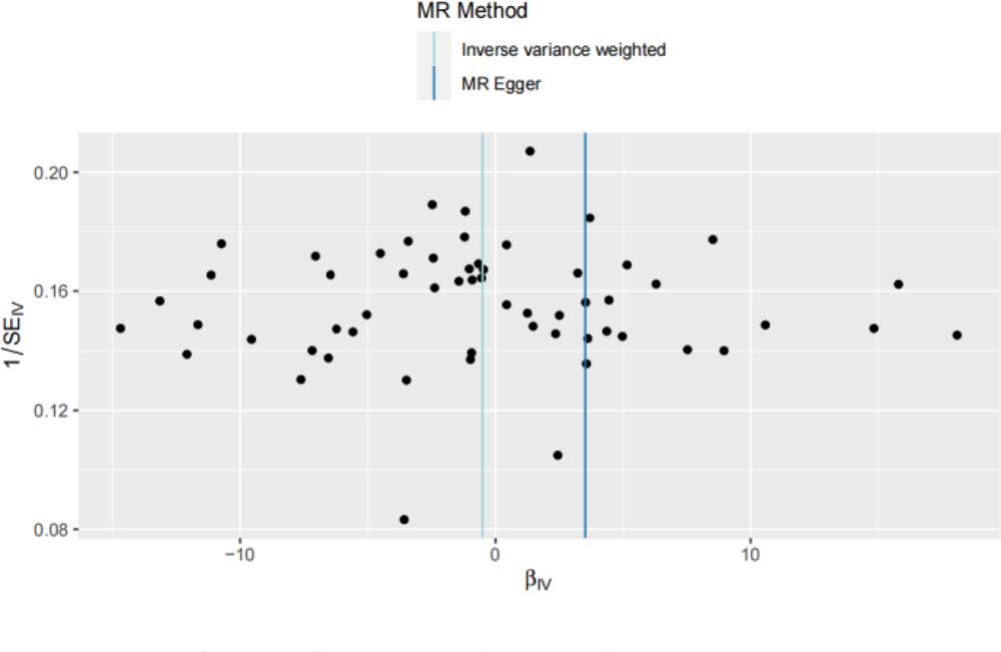
A funnel plot of the MR analysis from depressed affect to POI. The funnel plot shows a roughly symmetrical distribution on both sides, indicating a low likelihood of being influenced by potential bias. MR = Mendelian randomization, POI = premature ovarian insufficiency.

**Figure 5.**
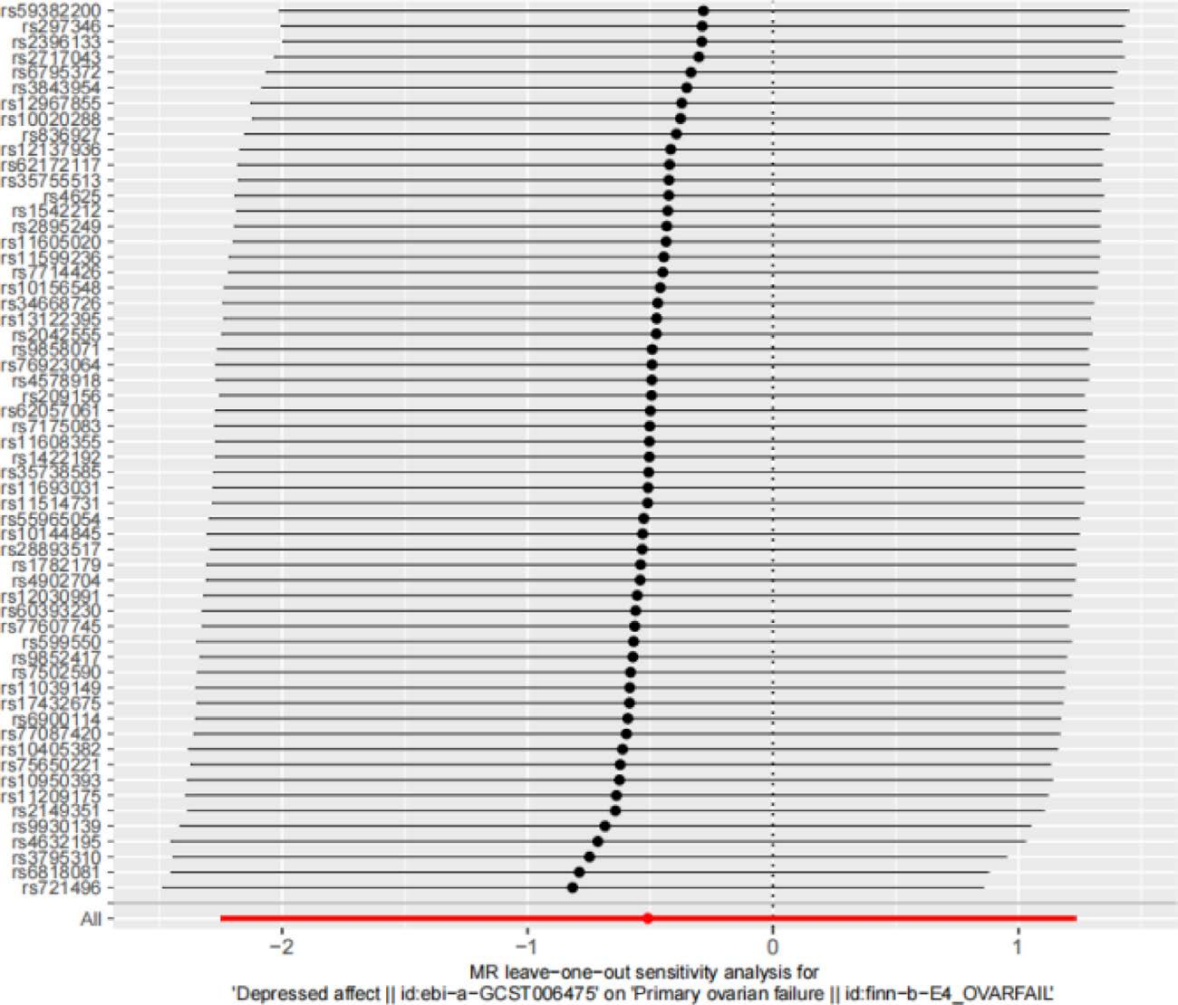
The leave-one-out sensitivity analysis from depressed affect to POI. The results show that after sequentially excluding each individual SNP, the analysis results of the remaining 57 SNPs were similar to the IVW analysis results that included all SNPs, and all remained to the right of the null line, indicating that the results of this MR analysis are robust. IVW = inverse-variance weighted, MR = Mendelian randomization, POI = premature ovarian insufficiency, SNPs = single nucleotide polymorphisms.

The results of Cochran Q test showed *Q* = 64.148 and *P* = .240, indicating that there was no heterogeneity among the included SNPs. The MR-Egger intercept is −0.067 and *P* = .320, indicating that the result of causal effect analysis is not interfered by pleiotropy, and the exclusivity hypothesis can be considered valid. The funnel plot shows a rough symmetry on both sides (Fig. 4), indicating a low likelihood of being affected by potential bias. MR-PRESSO results showed that no outlier SNPs were detected (*P* = .252). The sensitivity analysis of the “leave-one-out” showed that after the elimination test one by one, single SNPs were eliminated in turn, and the results of the remaining 57 SNPs analysis were similar to those of the IVW analysis with all SNPs included, and they were all on the right side of the invalid line (Fig. 5), indicating that the results of this MR Analysis were robust.

### 3.4. MR results of POI and depressive mood

#### 3.4.1. Screened IVs

The classical parameters *P* < 5 × 10^−8^, *r*^2^ = 0.001, kb = 10,000 were used as screening conditions, and there was no POI GWAS data significantly related to exposure, so the parameters were adjusted to *P* < 5 × 10^−6^, *r*^2^ = 0.001, kb = 10,000 for calculation. A total of 13 SNPs were screened out to be significantly correlated with POI, and depressed GWAS data were retrieved and extracted. Among them, 2 SNPs were missing from depression GWAS data without palindrome sequence, and no outliers were obtained by MR-PRESSO test. Finally, a total of 11 SNPs were included, and the *F*-statistic value of these SNPs was all >10. It indicates that a weak variable is less likely.

### 3.5. MR results for both samples

**Figure 6.**
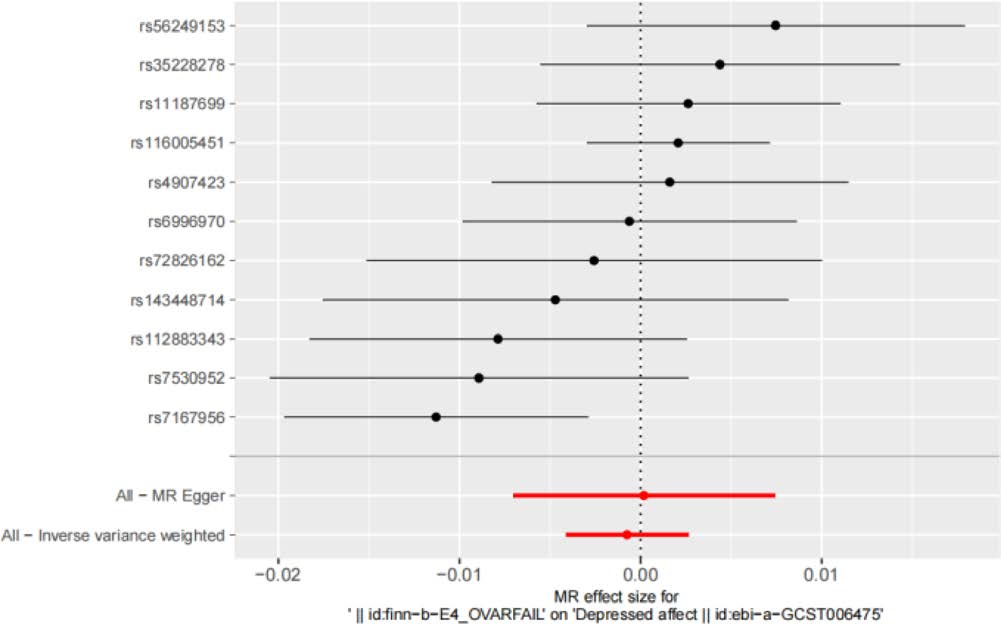
presents the MR analysis results for the associations from POI affect to depressed. affect using 2 different methods: IVW and MR Egger. IVW = inverse-variance weighted, MR = Mendelian randomization, POI = premature ovarian insufficiency.

**Figure 7.**
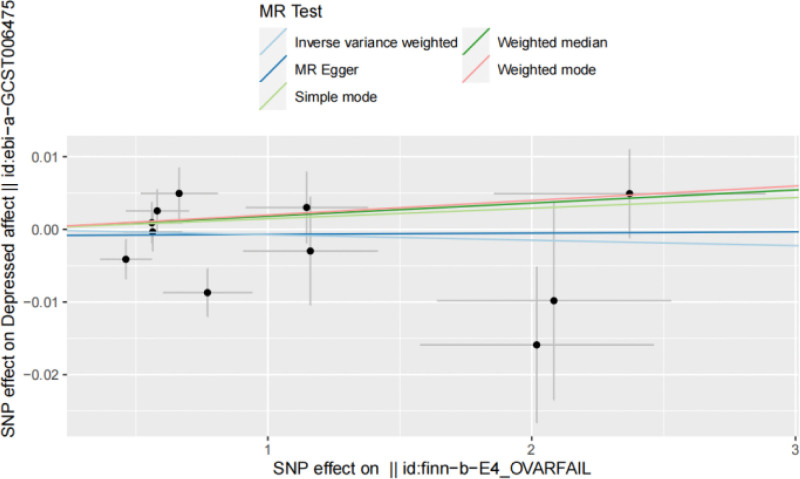
A scatter plot of the analysis results from POI affect to depressed using five different MR methods, with different colors representing different methods and dots representing different SNPs. MR = Mendelian randomization, POI = premature ovarian insufficiency, SNPs = single nucleotide polymorphisms.

IVW result: OR = 0.999, 95% CI: 0.996 to 1.003, *P* = .663, suggesting that POI may have nothing to do with depressed. The forest diagram of MR Analysis is shown in Fig. 6, and the MR-Egger method, as a supplement to the IVW results, also indicates that there is no obvious causal relationship between the 2. The relevant results of the five MR Testing methods are shown in Table 3, and the scatter diagram is shown in Fig. 7.

**Table 3:** The results of MR analysis from POI to depressed.

Method	nSNP	OR (95% CI)	*P*-value
MR Egger	11	1.000 (0.993–1.007)	.963
Weighted median	11	1.002 (0.999–1.006)	.350
Inverse variance weighted	11	0.999 (0.996–1.003)	.663
Simple mode	11	1.001 (0.992–1.008)	.682
Weighted mode	11	1.002 (0.992–1.006)	.381

CI = confidence interval, MR = Mendelian randomization, OR = odds ratio, POI = premature ovarian insufficiency, SNPs = single nucleotide polymorphisms.

### 3.6. Quality control

**Figure 8.**
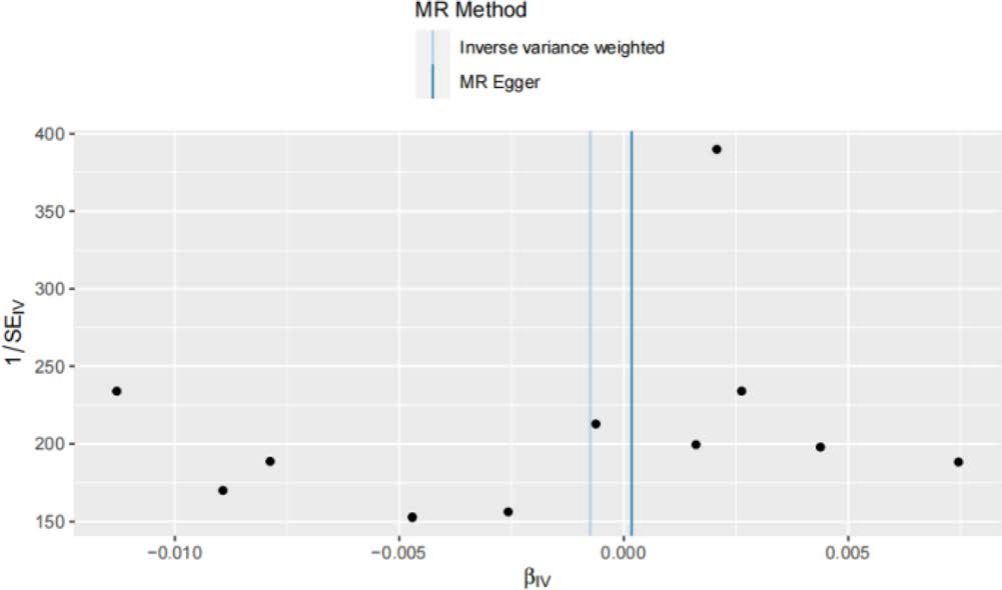
A funnel plot of the MR analysis from POI affect to depressed. The funnel plot shows a roughly symmetrical distribution on both sides, indicating a low likelihood of being influenced by potential bias. MR = Mendelian randomization, POI = premature ovarian insufficiency.

**Figure 9.**
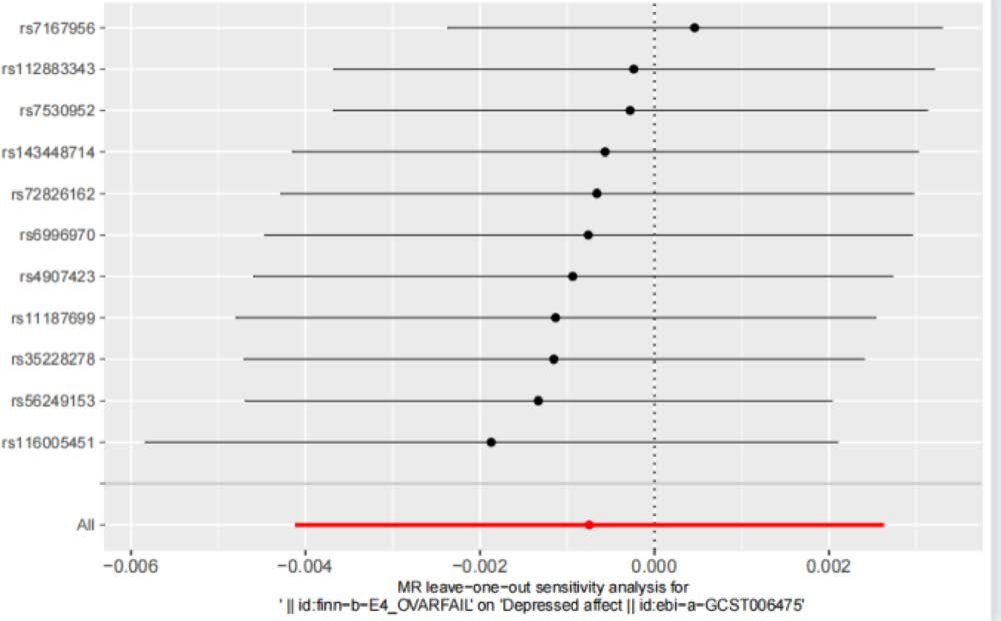
The leave-one-out sensitivity analysis from POI affect to depressed. The results indicate that after removing rs7167956, the *β* value crossed the null line, suggesting potential heterogeneity. The MR results remained robust after excluding the other SNPs. MR = Mendelian randomization, POI = premature ovarian insufficiency, SNPs = single nucleotide polymorphisms.

Cochran Q test showed *Q* = 15.75 and *P* = .107, indicating that there was no heterogeneity among the included SNPs. The MR-Egger intercept is −0.001 and *P* = .780, indicating that the result of causal effect analysis is not interfered by pleiotropy, and the exclusivity hypothesis can be considered valid. The funnel plot shows a rough symmetry on both sides (Fig. 8), indicating a low likelihood of being affected by potential bias. MR-PRESSO results showed that no outlier SNPs were detected (*P* = .128). The “leave-one-out” sensitivity analysis showed that the Beta value after rs7167956 exclusion crossed the zero line, suggesting potential heterogeneity, and the MR Results after the exclusion of the remaining SNPs were robust (Fig. 9).

## 4. Discussion

This study verified the correlation between depressed and POI for the first time through 2-way 2-sample MR analysis. IVW method, MR-Egger regression, WM, WME, and simple mode method showed that depressed did not increase the risk of POI; meanwhile, reverse MR Analysis also showed that there was no causal relationship between the 2.

Depressed mood almost intersperses the life cycle of the human body, and is a common mental illness, usually appearing in the early stage of life, and is easy to progress to depression and bipolar disorder.^[22]^ Nearly 25% of women have experienced major depressive episode in their lifetime, twice the rate of male major depressive episode, and the recurrence rate is as high as 60%.^[23]^ At the same time, depressed mood will greatly affect health status, leading to immune metabolic diseases, chronic pain, neurodegeneration and other diseases, and seriously endangering women’s physical and mental health as well as social and economic development.^[24]^ For women, multiple pressures from work, life, emotions and other aspects make women prone to depressed, which will affect the hypothalamic-pituitary-gonad axis and abnormal secretion of sex hormones.^[25]^ The hypothalamic-pituitary-gonadal axis is a multistage hormone system that regulates the secretion of female ovarian hormones through multiple feedback mechanisms of the ovary, pituitary gland and brain. The hypothalamic gonadotropin-releasing hormone induces the release of luteinizing hormone and follicle-stimulating hormone in the anterior pituitary, and further induces the release of progesterone (P) and estradiol (E_2_).^[26]^ Depressed mood will reduce the contents of norephrinephrine and 5-hydroxyindoleacetic acid in brain tissue through acute and chronic psychological stress, and inhibit the reproductive physiological function of the ovary. It leads to the disappearance of the sexual cycle and decreases the levels of follicle-stimulating hormone and E_2_, thus inducing the occurrence of POI.^[27]^ However, other studies have found that women with depressed mood and reproductive related mood disorders usually have normal ovarian hormone levels,^[28]^ indicating that depressed mood may not be directly involved in female reproductive endocrine,^[29]^ and there is no correlation between the 2. Although this study found no causal relationship between depressed and POI, the importance of depressed in the pathogenesis of POI should still be paid attention to.

Due to the high gonadotropin and low estrogen levels in POI patients, they often show abnormal menstruation, hot flushes, sweating, vaginal dryness, irritability, insomnia, decreased sexual desire and infertility, etc. In addition to their physiological changes, their depression is also affected by many social factors such as work, family and interpersonal relationships. Relevant studies have shown that the risk of depression associated with POI is 3.33 times higher than that of normal people (OR = 3.33, 95% CI = 2.31–4.81, *P* < .01),^[30]^ and about 43% of POI patients have depressed mood. The fluctuation of estrogen and progesterone will affect the generation and regulation of depressed mood, while the low hormone level of POI patients reduces the brain elastic adaptation to depressed mood, thus leading to the further aggravation of depression. Meanwhile, the further aggravation of depression will also lead to the progression of POI disease, and the 2 will interact to form a vicious circle.^[31,32]^ However, there is also evidence that hormone fluctuations do not cause depression in POI patients.^[10]^ Through MR Analysis, this study found no causal relationship between POI and depressed mood from the perspective of genetics, which is different from existing observational studies to some extent. The possible reasons for this result are as follows: the generation of depressed mood is complex and can be self-regulated within the range of human elastic adaptation through multiple factors and ways, and when it exceeds the elastic adaptation, it will be alleviated by other internal and external factors, which will not necessarily further aggravate the progress of depression or other mental diseases; although POI patients have a higher probability of depressed mood than normal people, it does not necessarily lead to the occurrence and progression of depression; the GWAS data used in this study were all from European populations, and the relevant GWAS data were few and the population was limited. This study is statistical and cannot further explore the mechanism. Follow-up studies need to be carried out among different ethnic groups. It is expected that more GWAS data can be published in the future to explore the causal effect between relevant exposure factors and diseases.

## 5. Conclusion

In summary, this study adopts the 2-way 2-sample MR method to explore the causal relationship between depressed and POI, and the results show that genetics cannot explain the causal relationship between depressed and POI. Nevertheless, public health departments and clinical workers still need to pay attention to the relationship between emotional factors and diseases, so as to alleviate patients’ adverse emotions. And improve the quality of life.

## Acknowledgments

Kan Chen conceived and wrote the paper, Li Wan, Xiaorong Ma, searched the related data, Xinmei Wang, Yanru Chen carried out statistical analysis, Lu Han reviewed the manuscript. All authors reviewed the manuscript. Thanks to all the authors who contributed to this article.

## Author contributions

**Data curation:** Kan Chen, Li Wan, Xiaorong Ma, Yanru Chen.

**Funding acquisition:** Lu Han.

**Investigation:** Li Wan, Xiaorong Ma.

**Methodology:** Xinmei Wang, Yanru Chen.

**Software:** Xinmei Wang, Yanru Chen.

**Visualization:** Kan Chen, Xinmei Wang.

**Writing—original draft:** Kan Chen.

**Writing—review & editing:** Lu Han.
